# Ecological Context Constrains Host‐Associated Genetic Structure in the Common Cuckoo

**DOI:** 10.1002/ece3.74396

**Published:** 2026-09-17

**Authors:** Jun‐Seo Go, Seongho Yun, Hae‐Ni Kim, Sue‐Jeong Jin, Myeong‐Chan Cha, Heesoo Lee, Jin‐Won Lee

**Affiliations:** ^1^ Department of Biology, Korea Institute of Ornithology Kyung Hee University Seoul Republic of Korea; ^2^ Bird Research SaeZiP Yangpyeong Republic of Korea

**Keywords:** avian brood parasites, habitat imprinting, host imprinting, host specialization, natal philopatry, population genetic structure

## Abstract

Host specialization is often expected to promote population genetic structure by restricting gene flow among individuals associated with different hosts. However, whether behavioral host specificity consistently generates spatial genetic differentiation remains unclear. The common cuckoo ( *Cuculus canorus* ), an obligate brood parasite, exhibits host specialization at the individual level, and several behavioral mechanisms—including natal philopatry, habitat imprinting, and host imprinting—have been proposed to explain its origin. In South Korea, cuckoos show a pronounced regional shift in host use, parasitizing the vinous‐throated parrotbill (*Suthora webbiana*) on the mainland and the meadow bunting ( *Emberiza cioides* ) on Jeju Island. We examined whether population genetic structure was associated with geographic distance, habitat composition, or host distribution in this system. Genetic structure was exceptionally weak across the mainland, and genetic relatedness showed no meaningful association with geographic distance, habitat composition, or predicted host distribution. Detectable differentiation was largely restricted to the geographically isolated Jeju population, with an additional deviation at Ansan that may reflect sampling heterogeneity rather than stable population structure. Despite ecological heterogeneity and the presence of multiple potential host species on the mainland, genetic variation was largely homogenized across the continuous mainland landscape. This pattern is consistent with extensive gene flow across the mainland, where *S. webbiana* is widespread and constitutes the predominant host. Our findings show that host specialization alone does not necessarily generate spatial genetic structure and highlight how ecological context and host dominance can constrain host‐associated genetic differentiation in highly mobile brood parasites.

## Graphical abstract

Host specialization is often expected to generate population genetic structure, yet this relationship remains unclear in highly mobile species. Using the common cuckoo in South Korea, we show that genetic structure is exceptionally weak across the mainland despite ecological heterogeneity and multiple host species, with differentiation emerging only under geographic isolation. These findings highlight how ecological context and host dominance can constrain host‐associated genetic differentiation.

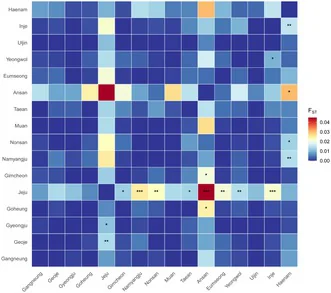

## 1 Introduction

Population genetic structure is shaped by a wide range of evolutionary and ecological processes, including behavioral mechanisms that influence patterns of dispersal, settlement, and gene flow among populations (Avise 2000). In birds, variation in dispersal (Miller et al. 2012; Camacho et al. 2013), breeding behavior (MacDougall‐Shackleton and MacDougall‐Shackleton 2001; Uy et al. 2018), and habitat selection (Cheek et al. 2022) can substantially affect genetic connectivity, sometimes even at relatively fine spatial scales (Lee et al. 2010; Camacho et al. 2013; Graham et al. 2017). Because such behavioral processes are often difficult to observe directly, population genetic structure provides an integrative and indirect means of inferring the mechanisms underlying population differentiation.

Avian brood parasites offer a particularly informative system for examining how behavioral mechanisms shape population genetic structure. By laying their eggs in the nests of host species and relying entirely on hosts for parental care, brood parasites engage in coevolutionary interactions that promote the evolution of host specificity (Friedmann 1928; Rothstein 1990; Davies 2000; Payne and Sorensen 2005; Feeney et al. 2014; Medina and Langmore 2016). Host specificity—defined as the preferential exploitation of particular host species—occurs not only among species but also among individuals within a species (Marchetti et al. 1998; Gibbs et al. 2000; Fossøy et al. 2011, 2016). Because host specialization can influence where individuals search for nests, settle, and reproduce, it has the potential to shape patterns of gene flow and, under some conditions, generate population genetic structure.

The common cuckoo ( *Cuculus canorus* ) is a long‐distance migratory brood parasite that breeds widely across Eurasia and exploits a diverse array of host species (Moksnes and Røskaft 1995; Davies 2000; Payne and Sorensen 2005; Erritzøe et al. 2012). Despite extensive research on host use and host–parasite coevolution in this species, how host specificity is generated and maintained remains incompletely understood. Host specificity in cuckoos has traditionally been discussed primarily in relation to female‐limited traits, such as egg phenotype and laying behavior (Marchetti et al. 1998; Gibbs et al. 2000; Fossøy et al. 2011, 2016). Female cuckoos can be highly specialized on a single host in these traits (Šulc et al. 2022; Koleček et al. 2021). If both sexes were host‐faithful and mated assortatively by host, such fidelity could in principle promote reproductive isolation among host races, as in the brood‐parasitic Vidua finches, where host‐specific imprinting couples host use directly to mate choice (Sorenson et al. 2003). Host fidelity need not, however, translate into assortative mating. Host specificity in cuckoos may instead be expressed at stages other than pairing—during settlement, space use and habitat selection in males, and additionally during host‐nest searching in females (Teuschl et al. 1998; Davies et al. 2006; Fuisz and De Kort 2007)—so that mating can occur at random with respect to host even when both sexes are host‐faithful. Host‐associated patterns therefore need not be restricted to females alone, yet need not generate host‐associated genetic divergence. Understanding how these behavioral processes translate into patterns of population genetic structure remains a central challenge in the study of avian brood parasitism.

To explain host selection in common cuckoos, three major, non‐mutually exclusive hypotheses have been proposed (Moksnes and Røskaft 1995). The natal philopatry hypothesis posits that individuals return to breed near their natal area, such that patterns of host use largely reflect local host availability and nest detectability rather than active host choice (de Brooke and Davies 1991). Under this hypothesis, population genetic structure is expected to be associated primarily with geographic distance. The habitat imprinting hypothesis suggests that individuals preferentially settle in habitats similar to those experienced during early development, thereby encountering host species characteristic of those habitats (Moksnes and Røskaft 1995; Teuschl et al. 1998; Vogl et al. 2002; Koleček et al. 2020). Under this hypothesis, population genetic structure is expected to covary with habitat composition rather than with geographic distance or host distribution per se. In contrast, the host imprinting hypothesis proposes that individuals imprint on their foster host species during early development and subsequently preferentially parasitize the same host species as adults (Yang et al. 2018). This hypothesis predicts that population genetic structure may be associated with host distribution rather than with geography or habitat per se. These alternative hypotheses therefore generate contrasting predictions about how behavioral processes should be reflected in spatial patterns of genetic differentiation.

South Korea provides a particularly informative natural system for evaluating these predictions, as it encompasses both an extensive mainland and a geographically distinct island system. Previous studies have documented pronounced regional differences in host use by common cuckoos in South Korea: on the mainland, cuckoos predominantly parasitize the vinous‐throated parrotbill (*Suthora webbiana*) as well as the Daurian redstart (*Phoenicurus auroreus*) (Lee 2014), whereas on Jeju Island they primarily parasitize the meadow bunting ( *Emberiza cioides* ), where both mainland species do not breed (Lee et al. 2010; Lee, Kang, et al. 2023). The vinous‐throated parrotbill, in particular, discriminates against and rejects non‐mimetic eggs (Lee and Yoo 2004; Lee et al. 2005), providing evidence of a coevolved host–parasite relationship. In Korean cuckoos, this regional shift in host use is accompanied by corresponding differences in egg phenotype. Cuckoos parasitizing the principal mainland host, the vinous‐throated parrotbill, lay blue eggs that closely match those of their host, whereas cuckoos using the meadow bunting on Jeju lay whitish, non‐blue eggs that match the bunting's pale eggs. These differences in host use and egg phenotype are associated with distinct maternal (mitochondrial) lineages in the two regions (Lee et al. 2021; Lee, Kang, et al. 2023). Because eggshell background color is maternally inherited (Fossøy et al. 2016; Merondun et al. 2025), however, such differentiation is not necessarily expected to be reflected in the biparentally inherited microsatellite markers analyzed here. Nevertheless, the contrasting host associations of mainland and Jeju cuckoos provide a useful natural system for evaluating the alternative mechanisms of host specialization outlined above and their predicted patterns of nuclear genetic structure.

In this study, we investigated the population genetic structure of common cuckoos in South Korea to evaluate how patterns of genetic relatedness among individuals covary with geographic distance, habitat composition, and host distribution. By comparing observed genetic patterns with predictions derived from the natal philopatry, habitat imprinting, and host imprinting hypotheses, we aimed to assess the relative importance of alternative behavioral mechanisms underlying genetic structure associated with host use.

## 2 Materials and Methods

### 2.1 Fieldwork

**FIGURE 1:**
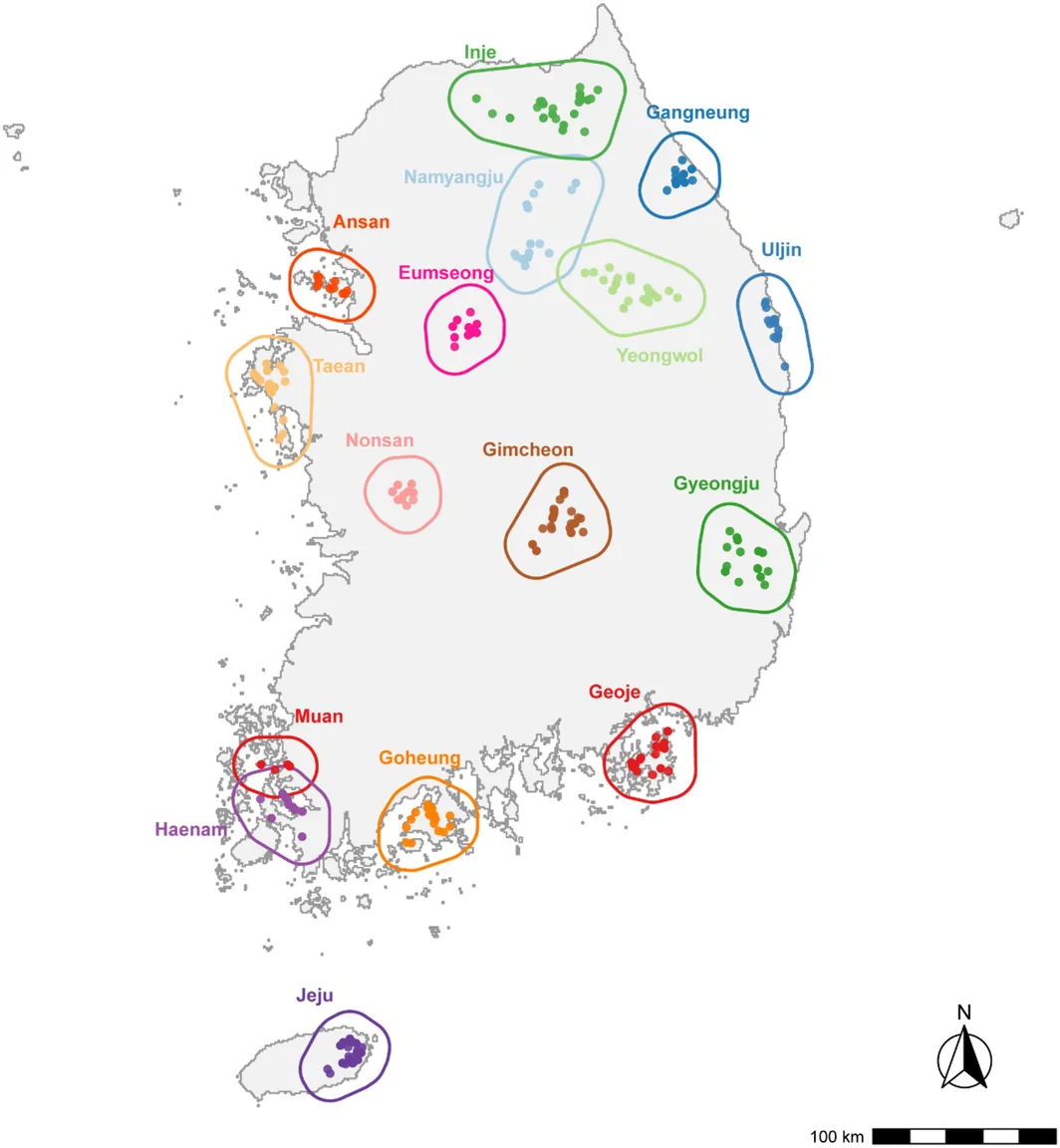
A map showing the capture locations of 364 common cuckoos collected from 16 populations across the Republic of Korea between 2021 and 2024. Sampling sites include 15 mainland populations and one insular population on Jeju Island.

We captured adult common cuckoos across South Korea during the breeding season (May–June) between 2021 and 2024 (Figure 1). Individuals were sampled from 16 populations, including 15 mainland sites and one insular population on Jeju Island, the largest island of South Korea (Figure 1). Mainland sites are located within a temperate climatic zone and are characterized by a mosaic of mountainous terrain, agricultural landscapes, and human settlements. In contrast, Jeju Island is a volcanic island off the southern coast of South Korea, where temperate and subtropical plant communities coexist.

In total, we captured 364 adult common cuckoos (331 males and 33 females) using mist nets in combination with playback of pre‐recorded male and female calls and dummy cuckoo models (Go et al. 2021, 2024; Lee et al. 2021; Lee, Kim, et al. 2023; Lee, Kang, et al. 2023). Sex was determined based on vocal cues (male: “cu‐coo,” female: bubbling call; Jin, Kim, et al. 2025; Jin, Lee, and Yoo 2025) and body size, following established criteria (Go et al. 2021). Each individual was fitted with a metal ring to avoid duplicate sampling, and capture coordinates (WGS84) were recorded using a smartphone‐based GPS. Blood samples (20–30 μL) were collected from the brachial wing vein and stored in 1.5‐mL tubes containing absolute ethanol, and kept at room temperature until further processing (Lee et al. 2021; Di Lecce et al. 2022). After sampling, all individuals were released at the site of capture. Cuckoo captures were conducted under permits issued by local governments in South Korea, and all field procedures complied with national legislation and the guidelines of the Kyung Hee University Animal Ethics Committee.

### 2.2 Genetic Data and Quality Control

Genomic DNA was extracted from blood samples using the DNeasy Blood and Tissue Kit (Qiagen, Hilden, Germany) following the manufacturer's instructions. Population genetic variation was characterized using microsatellite loci previously shown to be informative in the common cuckoo (Lee et al. 2021). Eleven loci were initially amplified (Ccμ 01, 09, 13, 60, 88, 100, 108, 119, 137, Clu 03, 05). PCR amplification and subsequent processing were performed by a commercial service provider (SolGent Co. Ltd., Daejeon, Republic of Korea). Amplification reactions were carried out in an ABI 9700 thermal cycler (Applied Biosystems) under the following conditions: an initial denaturation at 95°C for 15 min, followed by 35 cycles of denaturation at 95°C for 20 s, annealing at 54.0°C for 40 s, and extension at 72°C for 60 s, with a final extension at 72°C for 3 min. Fragment analysis was conducted on an ABI 3130XL DNA Analyzer (Applied Biosystems) using GeneScan 500 ROX size standards. Allele scores generated in GeneMapper v4.0 (Applied Biosystems) were independently reviewed by two analysts (J.‐W.L. and J.‐S.G.), with discrepancies resolved through cross‐validation to generate the final genotype dataset.

All loci were screened for linkage disequilibrium and Hardy–Weinberg equilibrium in Genepop v4.7 (Rousset 2008). No significant linkage disequilibrium was detected. Across loci, the number of alleles (N_A_) ranged from 3 to 22 and expected heterozygosity (H_E_) ranged from 0.04 to 0.92 (Table S1). After sequential Bonferroni correction, three loci (Ccu60, Clu03, and Clu05) deviated from Hardy–Weinberg equilibrium (Table S1). To assess potential genotyping artifacts, loci were screened using Micro‐Checker v2.2.3 (Van Oosterhout et al. 2004). No evidence of large‐allele dropout was detected. Signals consistent with potential null alleles was detected for Ccu60 at Geoje and for Clu05 at Goheung, but were not consistent across populations and did not affect multilocus results; therefore, these loci were retained. In contrast, Clu03 showed consistent stuttering and heterozygote deficits, and thus was excluded. A final set of 10 loci was therefore used in subsequent analyses.

### 2.3 Population Genetic Analyses

To evaluate whether close kinship among sampled individuals could bias estimates of population structure, we screened for potential sibships within populations using COLONY v.2.0 (Jones and Wang 2010; Lee et al. 2021). Sibship inference was conducted under a maximum‐likelihood framework assuming polygamy in both sexes and no inbreeding. One dyad exhibited a high posterior probability (≥ 0.95), but assignment confidence was low, and all individuals were retained for subsequent analyses.

Population structure was assessed using STRUCTURE v.2.3.4 (Pritchard et al. 2000; Falush et al. 2003; Evans et al. 2009; Bolton et al. 2016; Lee et al. 2016) under an admixture model with correlated allele frequencies. We evaluated *K* values from 1 to 10, with 10 independent runs per *K*. Each run consisted of a burn‐in of 100,000 iterations followed by 500,000 Markov Chain Monte Carlo (MCMC) iterations. The most likely number of clusters (*K*) was inferred based on mean model log‐likelihood values [Ln P(D)] across runs. Population‐level genetic differentiation was quantified using pairwise *F*_ST_ (Wright 1949) in Arlequin v.3.5 (Excoffier and Lischer 2010; Eberhart‐Phillips et al. 2015), with significance assessed by 10,000 permutations.

### 2.4 Geographic Distance, Habitat Composition, and Host Distribution

Pairwise geographic distances (km) among individuals were calculated from capture coordinates (WGS84) in SPAGeDi v.1.5 (Hardy and Vekemans 2002). To estimate genetic relationships among individuals, we calculated pairwise relatedness coefficients (*r*) for all pairs of individuals using the Queller and Goodnight (1989) estimator as implemented in SPAGeDi (Lee et al. 2010; Maher et al. 2017; Leedale et al. 2018). Relatedness estimates were computed using reference allele frequencies calculated from all sampled individuals. The resulting pairwise geographic distance and relatedness matrices were subsequently used in downstream analyses evaluating not only the relationship between geographic distance and genetic relatedness but also the effects of habitat composition and host distribution on genetic relatedness.

Habitat composition surrounding each capture location was quantified in QGIS v3.40.9 (QGIS Development Team 2026) using circular buffers of 2.5‐km radius. This radius was selected based on the approximate spatial extent of breeding‐season movements observed in our tracking data (unpublished) and was intended to characterize the broader habitat and host environment potentially available around each capture site, rather than to represent the specific area used by females for host‐nest searching or egg laying (e.g., Koleček et al. 2021). Land‐cover data were obtained from the Environmental Geographic Information Service (EGIS, https://egis.me.go.kr). National‐scale land‐cover maps were used to classify habitat types within each buffer. Among the available land‐cover categories, forest cover, grassland, and inland wetland were selected as the primary habitat types relevant to the habitat environment of the common cuckoo (Davies 2000; Williams et al. 2016; Moskát et al. 2019), as host composition and availability may differ among habitat types. Forest cover includes coniferous, deciduous, and mixed forests, whereas grassland comprises natural and artificial grassland. Inland wetlands include inland riparian vegetation areas and were defined as terrestrial habitats that remain consistently moist. For each buffer, the proportional area (%) of forest cover, grassland, and inland wetland was calculated relative to the total buffer area. These habitat composition metrics were subsequently used in statistical analyses to examine their relationships with genetic relatedness and population structure. Land‐cover data were not available for areas adjacent to the Korean Demilitarized Zone (DMZ); therefore, six individuals captured in these regions were excluded from the habitat composition analyses.

To examine the relationship between host distribution and population genetic structure, we generated host distribution indices using species distribution modeling. The vinous‐throated parrotbill, a primary host species in mainland, widely occurs in a broad range of habitats, including urban woodlands, forest edges, wetlands, and agricultural landscapes (Lee et al. 2010). In contrast, the meadow bunting, widely distributed in Jeju Island as well as mainland Korea, tends to be more closely associated with forest and grassland habitats (Deng et al. 2003). Because cuckoo populations parasitizing meadow buntings on Jeju Island are genetically differentiated from mainland populations, and cuckoos associated with the meadow bunting may occur at low frequency on the mainland (Lee et al. 2021), we focused on modeling the distribution of the meadow bunting to evaluate potential host‐associated genetic structure. We modeled the meadow bunting rather than the vinous‐throated parrotbill because the parrotbill is a widespread habitat generalist that occurs at high density across most of the mainland, offering little spatial contrast against which to test genetic variation, whereas variation in predicted meadow bunting distribution provides a more informative gradient of host availability. In order to predict the distribution of the meadow bunting, we conducted species distribution modeling using Maxent v3.4.4 (Phillips and Dudík 2008), which is well suited for modeling species distributions based on presence‐only occurrence data (Phillips et al. 2006; Phillips and Dudík 2008; Merow and Silander 2014). To reduce multicollinearity, highly correlated environmental variables (*r* > 0.8) were excluded based on pairwise Pearson correlation analyses (Elith et al. 2011; Dormann et al. 2013; Merow et al. 2013). As a result, eight environmental variables were retained for modeling, including seven bioclimatic variables (BIO2, BIO3, BIO4, BIO10, BIO12, BIO14, and BIO16) (Table S2) (Fick and Hijmans 2017) and one land‐cover variable from the ESA WorldCover 10 m 2021 dataset (Zanaga et al. 2022). Occurrence data for the meadow bunting were obtained from the 5th National Ecosystem Survey (2019–2023), conducted by the National Institute of Ecology (NIE 2024) under the Ministry of Environment, Republic of Korea. The compiled meadow bunting occurrence records used for modeling are shown, together with the predicted distribution, in Figure S2. To minimize potential spatial sampling bias and spatial autocorrelation, occurrence records were spatially thinned prior to modeling using the R package spThin (Aiello‐Lammens et al. 2015), applying a minimum nearest‐neighbor distance of 5 km. The thinning procedure was replicated 100 times, and the replicate retaining the largest number of occurrence records was selected for subsequent analyses. Occurrence records were randomly partitioned into training (70%) and testing (30%) sets. Models were run in Maxent using the subsample replication option with 10 replicate runs and a maximum of 5000 iterations (Phillips and Dudík 2008). Background points were generated within the study area using default settings, and model performance was evaluated using the area under the receiver operating characteristic curve (AUC) based on test data. The model achieved a mean test AUC of 0.651 ± 0.006 across ten replicate runs, indicating moderate discriminatory ability (Swets 1988). Such moderate AUC values are commonly observed for widely distributed species with broad environmental tolerances (McPherson and Jetz 2007; Lobo et al. 2008). Variable contribution analysis indicated that landcover showed the highest percent contribution (28.3%), followed by BIO10 (mean temperature of warmest quarter; 20.8%) and BIO2 (mean diurnal range; 20.2%). Permutation importance analysis identified BIO10 (31.3%) and BIO14 (precipitation of driest month; 21.2%) as the most influential predictors. The averaged logistic output grid across replicate runs was exported and processed in QGIS (Figure S2). Predicted habitat suitability values were extracted for each cuckoo sampling location and used as the predicted meadow bunting distribution index in subsequent analyses of genetic relatedness and population structure.

### 2.5 Statistical Analyses

Patterns of genetic differentiation were assessed using both population‐ and individual‐based approaches. Principal coordinate analysis (PCoA) based on the *F*_ST_ matrix was used to visualize genetic relationships among populations (Gower 1966; De Jong et al. 2019). Individual‐based analyses evaluated how geographic distance, habitat composition, and predicted meadow bunting distribution were associated with pairwise genetic relatedness. To test for isolation by distance (IBD), we examined how pairwise relatedness varied across geographic distance classes and constructed permutation‐derived 95% null envelopes based on spatial randomization (10,000 permutations) (Smouse and Peakall 1999; Lee et al. 2010). For visualization, geographic distances were divided into ten bins, and mean pairwise relatedness was summarized within each distance class. Because pairwise comparisons are not statistically independent, we applied maximum‐likelihood population‐effects (MLPE) mixed‐effects models, with pairwise relatedness as the response variable (Clarke et al. 2002; Row et al. 2017; Peterman and Pope 2021). Geographic distance, habitat composition, and predicted meadow bunting distribution were included as fixed‐effects, while individual identity was included as a random effect to account for repeated occurrences of individuals across pairwise comparisons. To assess the robustness of model‐based results and reduce reliance on linear model assumptions, permutation‐based Spearman's rank correlation tests were conducted as complementary analyses (Botero‐Delgadillo et al. 2020). These tests were based on 10,000 random permutations of predictor variables, and observed correlation coefficients were compared with null distributions generated under spatial randomization. Statistical significance was assessed at *α* = 0.05. All statistical analyses were performed in R v4.4.2 (R Core Team 2024).

## 3 Results

Genetic differentiation among common cuckoo populations in South Korea was generally low, indicating high overall genetic connectivity across the study area. STRUCTURE analyses consistently supported a single genetic cluster (*K* = 1; Figure S1), providing no evidence for strong hierarchical subdivision among the 16 sampled populations.

**FIGURE 2:**
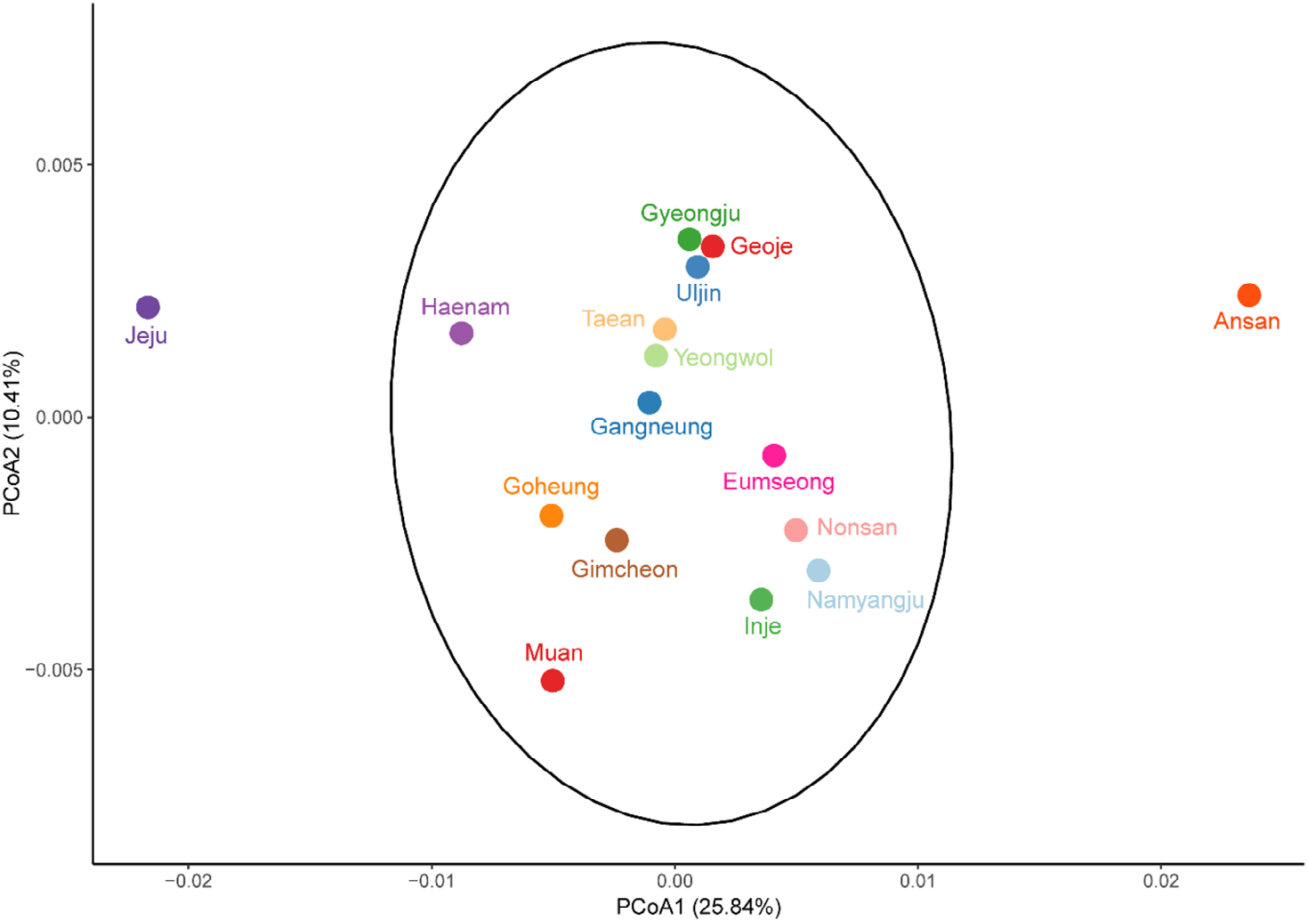
Principal coordinate analysis (PCoA) of pairwise *F*_ST_ values among 16 populations of the common cuckoo in South Korea. Most mainland populations form a single overlapping cluster, whereas the Jeju and Ansan populations are relatively displaced from the main cluster. The ellipse denotes the 95% confidence ellipse. The percentages of variation explained by the first two axes are shown in parentheses.

**FIGURE 3:**
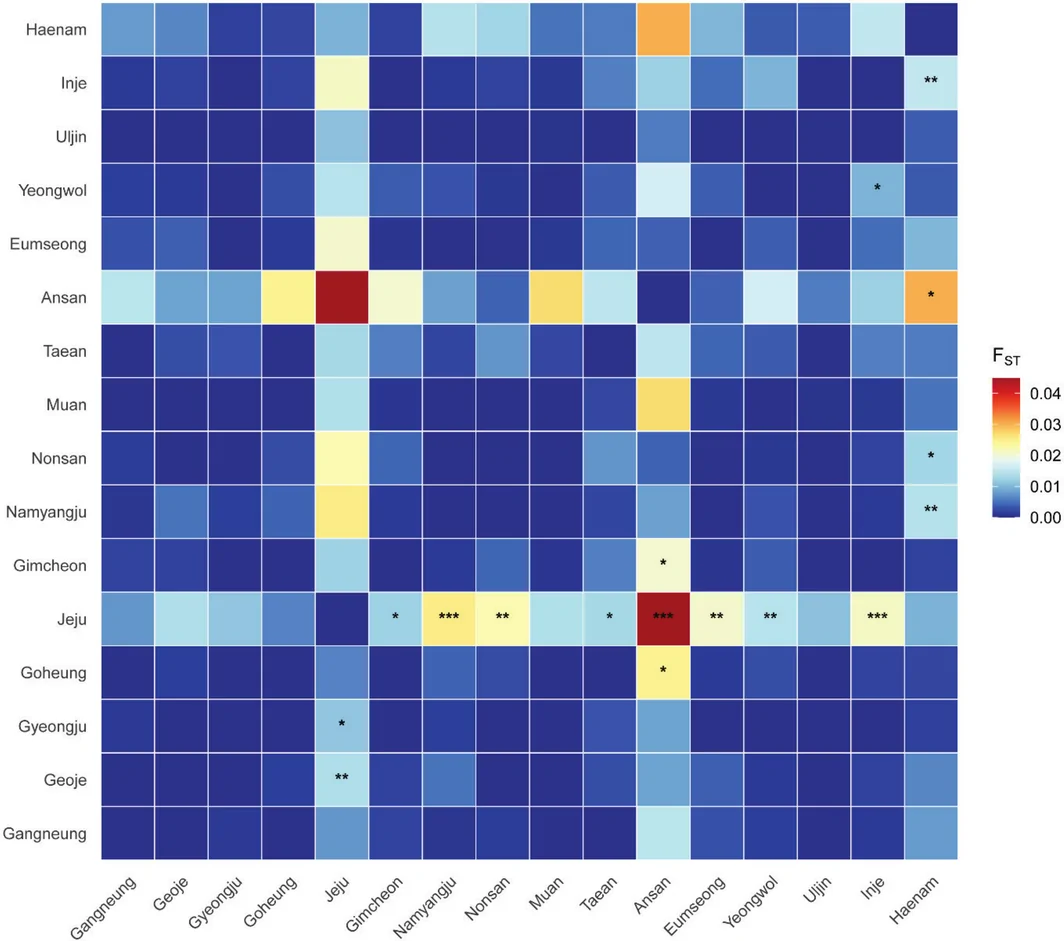
Heatmap of pairwise *F*_ST_ among 16 populations of the common cuckoo in South Korea. Warmer colors indicate higher genetic differentiation. Values in parentheses show pairwise *F*_ST_ estimates, and asterisks denote significance levels based on permutation tests (*p < 0.05, **p < 0.01, ***p < 0.001).

Despite this overall homogeneity, finer‐scale analyses revealed localized deviations from the general pattern. Principal coordinate analysis (PCoA) based on pairwise F_ST_ values showed that most mainland populations formed a broad, overlapping cluster, whereas individuals from Jeju Island and Ansan were consistently displaced from this cluster (Figure 2). The first two PCoA axes accounted for 25.84% and 10.41% of the total genetic variation, respectively. Pairwise *F*_ST_ values mirrored this pattern (Figure 3). While differentiation among mainland populations was generally low, elevated *F*_ST_ values occurred primarily in comparisons involving Jeju or Ansan, with the strongest differentiation observed between Jeju and Ansan (*F*_ST_ = 0.045). Together, these results indicate that genetic structure in South Korea is characterized by overall homogeneity punctuated by localized differentiation involving Jeju Island and Ansan.

**FIGURE 4:**
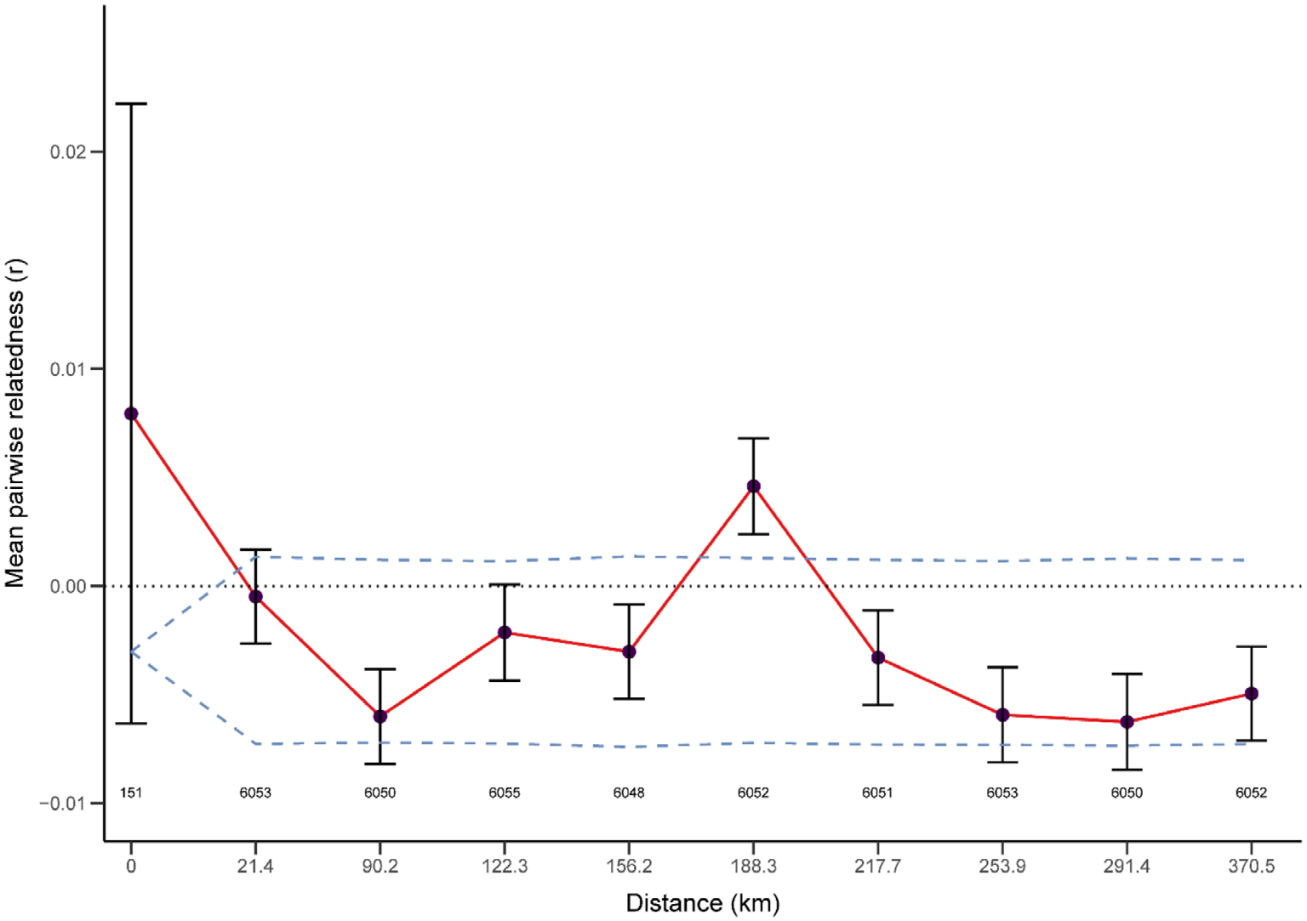
Mean pairwise relatedness across geographic distance classes. Each point represents the mean relatedness within a distance bin, with vertical bars showing standard errors. The dotted lines denote the 95% permutation‐derived null envelope.

**FIGURE 5:**
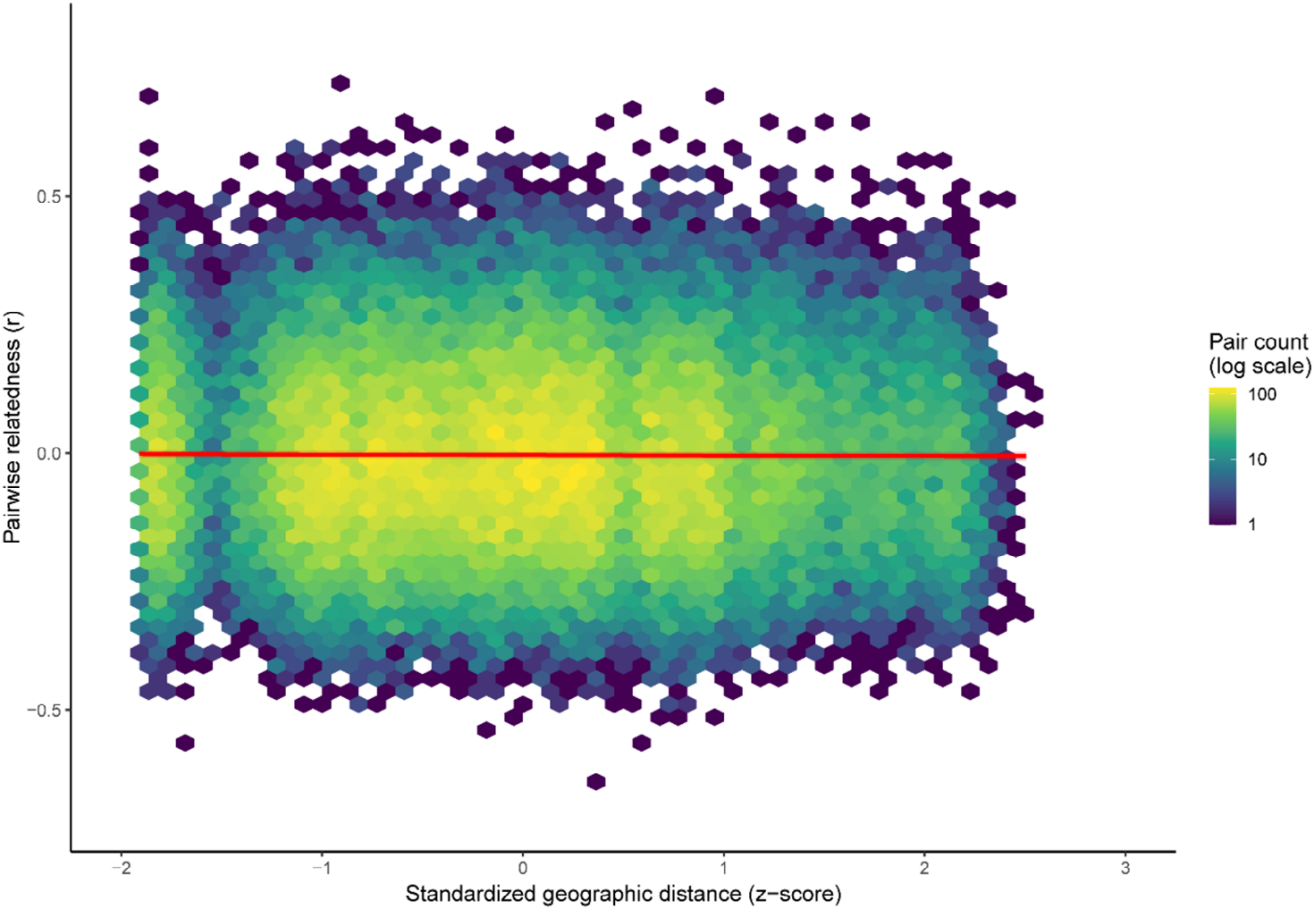
Effect of geographic distance on pairwise relatedness estimated using an MLPE model. Hexagonal color density indicates the distribution of pairwise relatedness values. The red line represents the MLPE fixed‐effects prediction across standardized geographic distance, where distance values were centered and scaled to *z*‐scores (mean = 0, SD = 1) prior to analysis.

To evaluate whether geographic separation contributes to genetic structuring, we examined the relationship between pairwise genetic relatedness and geographic distance. Among mainland populations, genetic relatedness showed no systematic change across geographic distance classes, with empirical estimates largely overlapping expectations under spatial randomization (Figure 4). Mixed‐effects models likewise detected no meaningful association between genetic relatedness and geographic distance (Table 1, Figure 5). When individuals from Jeju Island were included, geographic distance showed a statistically significant association with genetic relatedness; however, the estimated effect size was extremely small, indicating a negligible contribution of distance to variation in relatedness (Table 1). This weak pattern was largely driven by comparisons involving the geographically isolated Jeju population rather than by a general isolation‐by‐distance pattern across the mainland. Permutation‐based analyses likewise confirmed that the association between geographic distance and genetic relatedness was very weak (Table 2).

**TABLE 1:** Effects of geographic distance, habitat composition, and predicted host distribution on pairwise genetic relatedness based on mixed‐effects MLPE models.

Group	Predictor	Mainland only *β*	SE	*p*	Mainland + Jeju Island *β*	SE	*p*
All individuals	Geographic distance	−0.009	0.001	0.277	−0.004	0.001	< 0.001
	Forest proportion	0.000	0.001	0.983	0.000	0.001	0.513
	Grassland proportion	−0.002	0.001	0.013	−0.003	0.001	0.005
	Inland wetland proportion	0.003	0.002	0.058	0.003	0.001	0.027
	Predicted meadow bunting distribution	0.001	0.001	0.306	0.002	0.001	0.070
Female only	Geographic distance	0.001	0.008	0.943	0.005	0.007	0.551
	Forest proportion	−0.017	0.008	0.036	−0.016	0.008	0.036
	Grassland proportion	−0.009	0.009	0.313	−0.015	0.008	0.054
	Inland wetland proportion	0.011	0.008	0.194	0.015	0.008	0.050
	Predicted meadow bunting distribution	0.007	0.010	0.488	0.006	0.008	0.506

*Note:* Estimates (*β*), standard errors (SE), and *p*‐values are presented for analyses of all individuals and female individuals, conducted for mainland populations only and for mainland and Jeju Island combined.

**TABLE 2:** Spearman's rank correlations between pairwise genetic relatedness and geographic distance, habitat composition, and predicted host distribution.

Group	Predictor	Mainland only *ρ*	*p*	Mainland + Jeju Island *ρ*	*p*
All individuals	Geographic distance	−0.007	0.122	−0.018	< 0.001
	Forest proportion	−0.007	0.100	−0.017	< 0.001
	Grassland proportion	−0.023	0.001	−0.015	< 0.001
	Inland wetland proportion	−0.013	0.003	−0.015	< 0.001
	Predicted meadow bunting distribution	−0.007	0.122	−0.017	< 0.001
Female only	Geographic distance	0.031	0.496	0.021	0.622
	Forest proportion	−0.094	0.043	−0.078	0.076
	Grassland proportion	−0.038	0.412	−0.070	0.103
	Inland wetland proportion	0.001	0.980	0.039	0.370
	Predicted meadow bunting distribution	0.032	0.496	0.021	0.622

*Note:* Spearman's correlation coefficients (*ρ*) and permutation‐based *p*‐values are presented for analyses of all individuals and female individuals, conducted for mainland populations only and for mainland and Jeju Island combined.

**FIGURE 6:**
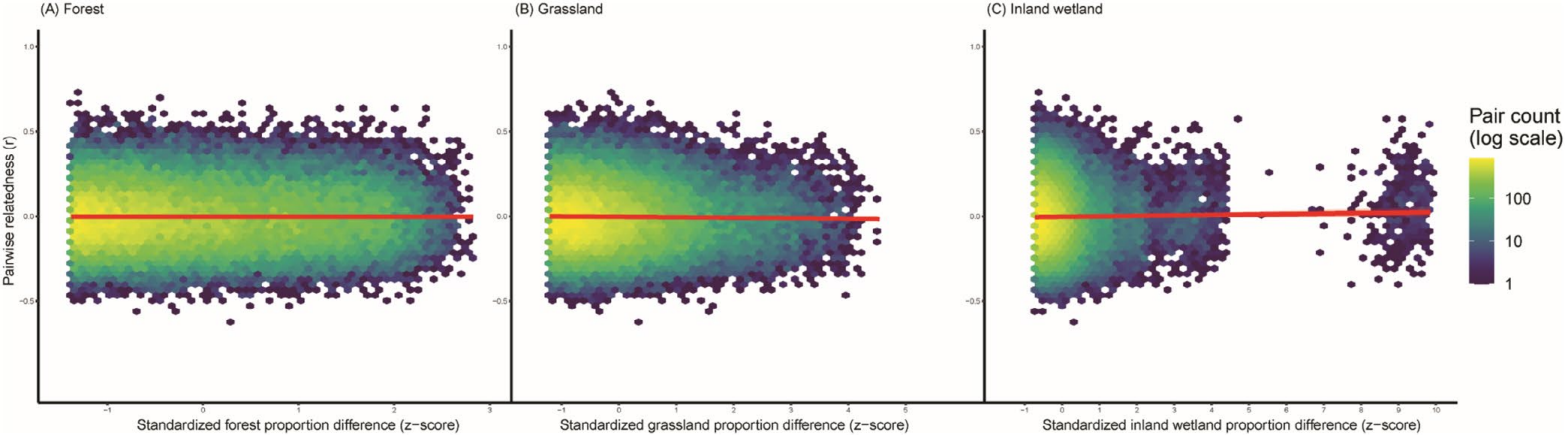
Effects of habitat composition differences on pairwise relatedness estimated using MLPE models. Panels show the relationships between pairwise relatedness and standardized absolute differences (*z*‐scored; mean = 0, SD = 1) in habitat proportions between paired individuals for (A) grassland, (B) forest, and (C) inland wetland. Hexagonal color density indicates the distribution of pairwise relatedness values (log‐scaled pair counts). The red line represents the MLPE fixed‐effects prediction across each standardized habitat difference.

We next assessed whether local habitat composition was associated with patterns of genetic relatedness. Across mainland populations, habitat variables showed little explanatory power. Forest cover showed no association with genetic relatedness (Table 1, Figure 6), whereas grassland proportion showed a statistically detectable but extremely small negative effect (*β* ≈ −0.0025; Table 1, Figure 6). Inland wetland proportion showed no consistent association (Table 1, Figure 6). Including individuals from Jeju Island did not qualitatively alter these results: some habitat variables reached statistical significance in model‐based analyses, but the estimated effect sizes remained negligible (Table 1). Overall, habitat composition made only a minimal contribution to genetic structuring across populations. Permutation‐based analyses yielded patterns consistent with the MLPE models (Table 2). Although several predictors reached statistical significance—particularly when Jeju Island individuals were included—the corresponding correlation coefficients were very close to zero, indicating that these relationships were extremely weak.

**FIGURE 7:**
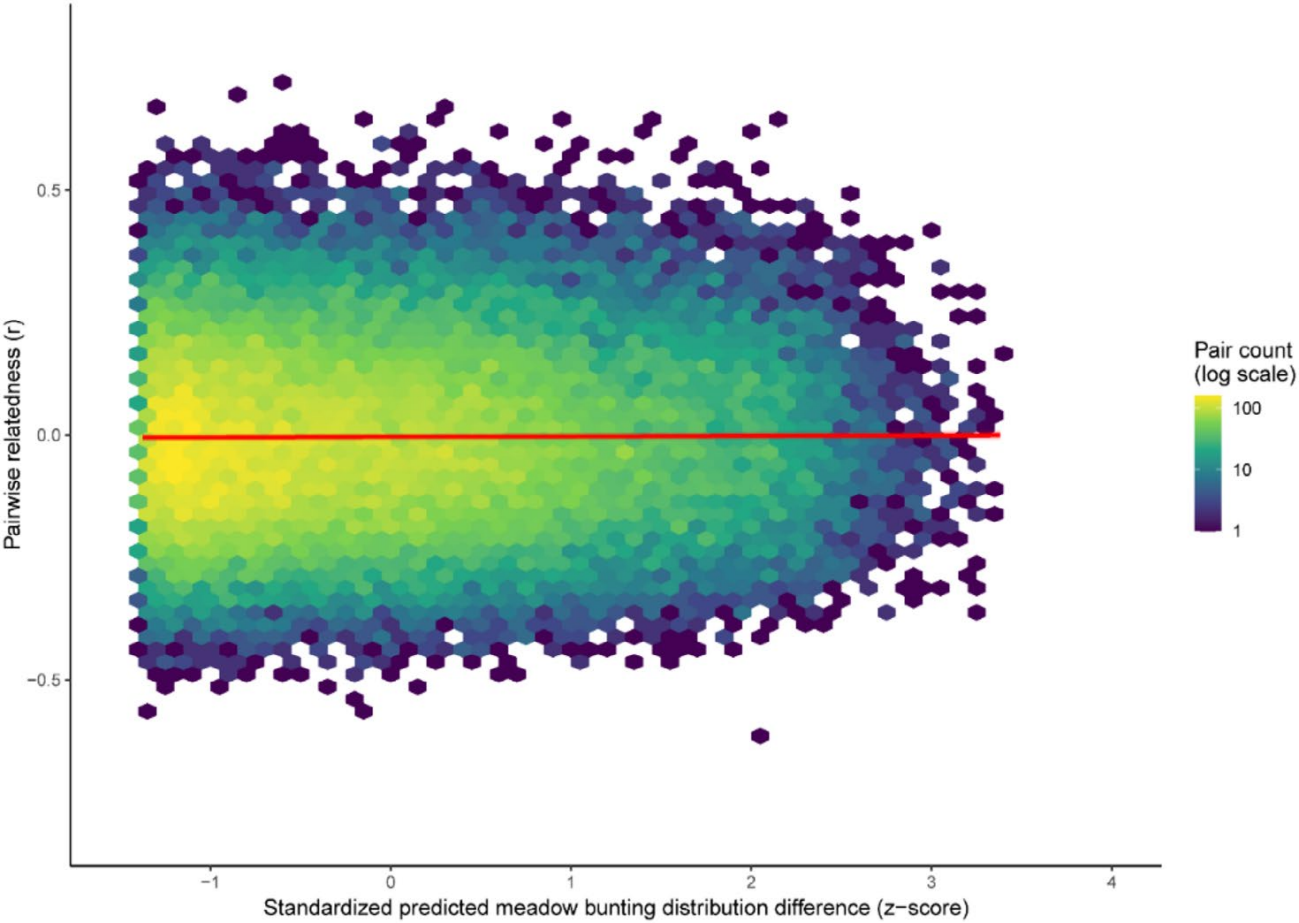
Effect of predicted meadow bunting distribution difference on pairwise genetic relatedness estimated using an MLPE model. Hexagonal color density indicates the distribution of pairwise relatedness values. The red line represents the MLPE fixed‐effects prediction across standardized predicted meadow bunting distribution difference, defined as the *z*‐scored absolute difference in predicted meadow bunting distribution values between paired capture locations (mean = 0, SD = 1).

Finally, we examined whether spatial variation in host distribution was associated with genetic relatedness. Predicted meadow bunting distribution showed no detectable relationship with genetic relatedness among cuckoos, either among mainland populations (Table 1, Figure 7) or when individuals from Jeju Island were included (Table 1). Thus, spatial variation in predicted host distribution did not explain variation in genetic relatedness.

Analyses restricted to 33 female individuals yielded results qualitatively consistent with those obtained from the full dataset. No meaningful associations were detected between genetic relatedness and geographic distance or predicted host distribution. Among habitat composition, forest cover showed a statistically detectable but extremely small negative effect (*β* ≈ −0.017; Table 1). Exclusion of the single female individual from Jeju Island did not qualitatively change these results (Table 1). Overall, these analyses provide no evidence that spatial variation in host distribution explains patterns of genetic relatedness in South Korean common cuckoos.

## 4 Discussion

Population genetic analyses revealed that common cuckoos breeding in South Korea exhibit exceptionally weak population genetic structure. Despite sampling across a broad geographic range encompassing both the mainland and Jeju Island, genetic variation was largely homogenized across populations, with no clear hierarchical subdivision detected. This overall pattern indicates extensive gene flow operating across the Korean Peninsula, consistent with the high dispersal ability and migratory behavior of this species (Willemoes et al. 2014; Hewson et al. 2016; Vega et al. 2016; Lee, Kang, et al. 2023; Sokolov et al. 2024; Cheng et al. 2025).

Against this backdrop of genetic homogeneity, localized deviations were consistently detected for both Jeju Island and Ansan. The distinctiveness of the Jeju population is particularly notable given its geographic isolation and pronounced shift in dominant host use. Whereas mainland populations primarily parasitize the vinous‐throated parrotbill, cuckoos on Jeju Island predominantly exploit the meadow bunting (Lee et al. 2021; Lee, Kang, et al. 2023). The vinous‐throated parrotbill is absent from Jeju, where cuckoos parasitize the meadow bunting and form a distinct maternal (mitochondrial) lineage relative to the mainland (Lee et al. 2021; Lee, Kang, et al. 2023), which may reflect host‐associated differentiation at the maternal level. Because host use and geographic isolation are confounded on Jeju, however, the modest nuclear differentiation of this population cannot be attributed to host use alone. Notably, Lee et al. (2021) found no comparable mainland–Jeju differentiation in the lesser cuckoo, which experiences the same geographic separation but uses the same principal host in both regions. Although species‐specific differences in dispersal or demographic history cannot be excluded, this contrast suggests that island isolation alone may not fully explain the differentiation observed in common cuckoos. Instead, geographic isolation may interact with regionally structured host use to restrict gene flow and promote genetic differentiation, and extensive connectivity across the mainland may prevent comparable divergence from emerging.

In contrast, the elevated differentiation involving Ansan may reflect sampling‐related factors rather than stable population structure. Fieldwork in this region was conducted early in the breeding season (10–14 May, in both 2021 and 2022), raising the possibility that transient individuals passing through the area were included rather than locally established breeders. Ansan is also located along the western coast of the Korean Peninsula, a region that may lie along spring migratory routes, meaning that individuals captured at this site may represent a mixture of birds originating from multiple breeding regions. Such temporal and geographic heterogeneity in sampling can inflate estimates of genetic differentiation without reflecting persistent population structure (Gregory et al. 2023).

Across the mainland, neither host distribution, habitat composition, nor geographic distance explained patterns of genetic relatedness. Predicted spatial variation in meadow bunting distribution showed no association with genetic relatedness, and habitat composition variables exhibited at most weak and biologically negligible effects. Similarly, geographic distance did not structure genetic relatedness within the mainland, providing no support for the spatial genetic pattern expected under strong natal philopatry at this scale. Taken together, these findings provide little support for the predicted genetic patterns of natal philopatry, habitat imprinting, or host imprinting under the ecological conditions prevailing across the South Korean mainland.

The absence of host‐associated genetic structure within the mainland is particularly informative. Although meadow buntings occur across both the mainland and Jeju Island, the vinous‐throated parrotbill is widely distributed at high densities across the mainland and occupies a broad range of habitats as a habitat generalist (Lee et al. 2010). Under such conditions, the widespread availability of a predominant host may reduce opportunities for spatial segregation among cuckoos associated with different hosts. When one principal host is abundant and broadly distributed, host‐associated differences in settlement or space use may not translate into geographically structured gene flow (Lee et al. 2021). Host specificity may therefore persist at the level of individual behavior or maternal lineage, yet fail to manifest as spatial genetic structure.

These findings suggest that the emergence of population genetic structure in brood parasites depends not only on host specialization itself, but also on the ecological context in which host lineages interact. In highly mobile species such as the common cuckoo, extensive dispersal can rapidly homogenize genetic variation across continuous landscapes. Under such conditions, behavioral specialization in host use may persist without generating detectable spatial genetic differentiation. In contrast, population‐level divergence is more likely to arise where ecological discontinuities or geographic barriers restrict gene flow, as suggested by the weak differentiation observed on geographically isolated Jeju Island.

A further consideration is that our habitat‐ and host‐based predictors are indirect: predicted meadow bunting distribution and land‐cover composition are landscape‐level proxies, and because we did not identify the actual host species used by each genotyped individual, a direct host‐based test at the individual level was not possible. At the same time, it is important to consider the resolution of the genetic markers used in the present study. Microsatellite loci are well suited for detecting population‐level genetic structure and have been widely applied in studies of dispersal and gene flow in birds (Wright et al. 2005; Genovart et al. 2013; Bertrand et al. 2014; Botero‐Delgadillo et al. 2020). However, their power to resolve subtle genetic differentiation associated with fine‐scale behavioral specialization may be limited, particularly in systems characterized by extensive gene flow. Host‐associated genetic differences may therefore persist at the level of individual lineages or specific genomic regions without being detectable in multilocus microsatellite data. Critically, host‐associated adaptation in cuckoos—most notably eggshell coloration and patterning—is inherited maternally, through the mitochondrial genome and the female‐specific W chromosome, so that maternally inherited markers (mitochondrial DNA and W‐linked loci) provide the most direct test for host‐associated lineages (Fossøy et al. 2016; Merondun et al. 2024, 2025). The extent to which host specificity extends to males remains debated: some studies infer that males mate across host races and homogenize the biparentally inherited nuclear genome (Gibbs et al. 2000; Merondun et al. 2025), whereas host‐specific dispersal in both sexes has been inferred for Korean common cuckoos (Lee et al. 2021). Our data do not distinguish between these alternatives: nuclear differentiation was detected between the mainland and Jeju, where host use and geographic isolation covary, but little structure was detected within the parrotbill‐dominated mainland. Under both scenarios, biparentally inherited microsatellites are expected to reveal host‐associated structure only weakly, so the nuclear homogeneity observed here is consistent with, rather than evidence against, host‐associated differentiation at the maternal level. Future studies employing genome‐wide approaches, such as RAD sequencing or whole‐genome resequencing, could provide higher resolution to test whether cryptic or locus‐specific patterns of host‐associated differentiation exist in this system.

In summary, our findings indicate that population genetic structure in common cuckoos is strongly shaped by the interaction between dispersal, ecological context, and host availability. Within South Korea, genetic variation is largely homogenized across the mainland despite ecological heterogeneity and the presence of multiple potential host species. Weak population differentiation was detected on Jeju Island, where geographic isolation coincides with a marked shift in dominant host use. On Jeju, however, geographic isolation and the shift in dominant host use are confounded, so the contribution of host specialization per se cannot be separated from that of island isolation. These results indicate that spatial variation in host use, habitat composition, and geographic distance does not necessarily translate into spatial genetic structure in highly mobile brood parasites, emphasizing the importance of considering both ecological dominance and dispersal processes when interpreting patterns of host‐associated differentiation.

## Author Contributions

**Jun‐Seo Go:** conceptualization (equal), data curation (lead), formal analysis (lead), investigation (equal), methodology (lead), resources (supporting), visualization (lead), writing – original draft (lead), writing – review and editing (equal). **Seongho Yun:** data curation (supporting), formal analysis (supporting), investigation (equal), methodology (supporting), resources (supporting), visualization (supporting). **Hae‐Ni Kim:** data curation (supporting), investigation (equal), resources (supporting). **Sue‐Jeong Jin:** data curation (supporting), investigation (equal), resources (supporting). **Myeong‐Chan Cha:** data curation (supporting), investigation (equal), resources (supporting). **Heesoo Lee:** data curation (supporting), investigation (equal), resources (supporting). **Jin‐Won Lee:** conceptualization (lead), formal analysis (supporting), funding acquisition (lead), investigation (equal), methodology (supporting), project administration (lead), resources (lead), supervision (lead), visualization (supporting), writing – original draft (supporting), writing – review and editing (equal).

## Funding

This work was supported by the National Research Foundation of Korea, NRF‐2020R1I1A2063567.

## Conflicts of Interest

The authors declare no conflicts of interest.

## Supporting information


**Table S1:** Summary statistics and quality‐control tests for 11 microsatellite loci screened in the common cuckoo. Results of Hardy–Weinberg equilibrium (HWE) and linkage disequilibrium (LD) tests are shown for the 11 microsatellite loci initially screened in the common cuckoo, together with the number of alleles per locus (*N*_A_), expected heterozygosity (*H*_E_), and within‐population inbreeding coefficient (*F*_IS_). Loci showing significant deviations from HWE are indicated by asterisks.
**Table S2:** Definitions of the seven bioclimatic variables (WorldClim) used in this study. Variables were selected to minimize collinearity while capturing temperature and precipitation gradients relevant to the study region.
**Figure S1:** STRUCTURE analysis of 364 common cuckoos ( *Cuculus canorus* ) genotyped at 10 microsatellite loci. (a) Mean log‐likelihood of the data [Ln *P*(*D*)] as a function of the assumed number of clusters (*K* = 1–16). (b, c) Individual admixture proportions at *K* = 2 and *K* = 3, respectively. Each vertical bar represents one individual, and individuals are grouped by sampling population.
**Figure S2:** Observed occurrence and predicted distribution of the meadow bunting ( *Emberiza cioides* ) in South Korea. (a) Compiled occurrence records (*n* = 1396) used for species distribution modeling. (b) Predicted habitat suitability from a Maxent model (mean of 10 replicate runs; values range from 0 = unsuitable to 1 = highly suitable). yellow colors indicate higher predicted suitability, whereas blue colors indicate lower predicted suitability.

**Data S1:** Microsatellite genotypes with ReadMe.

**Data S2:** Pairwise relatedness and geographic distance with ReadMe.

**Data S3:** Individual habitat proportions with ReadMe.

**Data S4:** Meadow bunting distribution index per individual with ReadMe.

## Data Availability

All the required data are uploaded as Supporting Information.

## References

[ece374396-bib-0001] Aiello‐Lammens, M. E. , R. A. Boria , A. Radosavljevic , B. Vilela , and R. P. Anderson . 2015. “spThin: An R Package for Spatial Thinning of Species Occurrence Records for Use in Ecological Niche Models.” Ecography 38: 541–545. 10.1111/ecog.01132.

[ece374396-bib-0002] Avise, J. C. 2000. Phylogeography: The History and Formation of Species. Harvard University Press.

[ece374396-bib-0003] Bertrand, J. , Y. Bourgeois , B. Delahaie , et al. 2014. “Extremely Reduced Dispersal and Gene Flow in an Island Bird.” Heredity 112: 190–196. 10.1038/hdy.2013.91. PMC3907105 24084644

[ece374396-bib-0004] Bolton, P. E. , A. J. West , A. P. Cardilini , et al. 2016. “Three Molecular Markers Show no Evidence of Population Genetic Structure in the Gouldian Finch ( *Erythrura gouldiae* ).” PLoS One 11: e0167723. 10.1371/journal.pone.0167723. PMC5147959 27936082

[ece374396-bib-0005] Botero‐Delgadillo, E. , V. Quirici , Y. Poblete , et al. 2020. “Range‐Wide Genetic Structure in the Thorn‐Tailed Rayadito Suggests Limited Gene Flow Towards Peripheral Populations.” Scientific Reports 10: 9409. 10.1038/s41598-020-66450-7. PMC7287099 32523081

[ece374396-bib-0006] Camacho, C. , D. Canal , and J. Potti . 2013. “Nonrandom Dispersal Drives Phenotypic Divergence Within a Bird Population.” Ecology and Evolution 3: 4841–4848. 10.1002/ece3.563 PMC3867915 24363908

[ece374396-bib-0007] Cheek, R. G. , B. R. Forester , P. E. Salerno , et al. 2022. “Habitat‐Linked Genetic Variation Supports Microgeographic Adaptive Divergence in an Island‐Endemic Bird Species.” Molecular Ecology 31: 2830–2846. 10.1111/mec.16438. PMC9325526 35315161

[ece374396-bib-0008] Cheng, Z. , D. Ma , M. Lu , X. Du , L. Kong , and X. Bao . 2025. “Variation of Migration Routes in the Central Asian‐Breeding Common Cuckoo Population Influenced by the Qinghai‐Tibet Plateau.” Avian Research 16: 100265. 10.1016/j.avrs.2025.100265.

[ece374396-bib-0009] Clarke, R. T. , P. Rothery , and A. F. Raybould . 2002. “Confidence Limits for Regression Relationships Between Distance Matrices: Estimating Gene Flow With Distance.” Journal of Agricultural, Biological, and Environmental Statistics 7: 361–372. 10.1198/108571102320.

[ece374396-bib-0010] Davies, N. B. 2000. Cuckoos, Cowbirds and Other Cheats. T & A D Poyser.

[ece374396-bib-0011] Davies, N. B. , J. Madden , S. Butchart , and J. Rutila . 2006. “A Host‐Race of the Cuckoo *Cuculus canorus* With Nestlings Attuned to the Parental Alarm Calls of the Host Species.” Proceedings of the Royal Society B: Biological Sciences 273: 693–699. 10.1098/rspb.2005.3324. PMC1560078 16608688

[ece374396-bib-0012] de Brooke, M. , and N. Davies . 1991. “A Failure to Demonstrate Host Imprinting in the Cuckoo ( *Cuculus canorus* ) and Alternative Hypotheses for the Maintenance of Egg Mimicry.” Ethology 89: 154–166. 10.1111/j.1439-0310.1991.tb00301.x.

[ece374396-bib-0013] De Jong, A. , O. Kleven , J. E. Østnes , et al. 2019. “Birds of Different Feather Flock Together‐Genetic Structure of Taiga Bean Goose in Central Scandinavia.” Bird Conservation International 29: 249–262. 10.1017/S0959270918000205.

[ece374396-bib-0014] Deng, W.‐H. , G.‐M. Zheng , and W. Gao . 2003. “Nesting Success of the Meadow Bunting Along Habitat Edges in Northeastern China.” Journal of Field Ornithology 74: 37–44. 10.1648/0273-8570-74.1.37.

[ece374396-bib-0015] Di Lecce, I. , J. Sudyka , D. F. Westneat , and M. Szulkin . 2022. “Preserving Avian Blood and DNA Sampled in the Wild: A Survey of Personal Experiences.” Ecology and Evolution 12: e9232. 10.1002/ece3.9232. PMC9424668 36052299

[ece374396-bib-0016] Dormann, C. F. , J. Elith , S. Bacher , et al. 2013. “Collinearity: A Review of Methods to Deal With It and a Simulation Study Evaluating Their Performance.” Ecography 36: 27–46. 10.1111/j.1600-0587.2012.07348.x.

[ece374396-bib-0017] Eberhart‐Phillips, L. J. , J. I. Hoffman , E. G. Brede , et al. 2015. “Contrasting Genetic Diversity and Population Structure Among Three Sympatric Madagascan Shorebirds: Parallels With Rarity, Endemism, and Dispersal.” Ecology and Evolution 5: 997–1010. 10.1002/ece3.1393. PMC4364815 25798218

[ece374396-bib-0018] Elith, J. , S. J. Phillips , T. Hastie , M. Dudík , Y. E. Chee , and C. J. Yates . 2011. “A Statistical Explanation of MaxEnt for Ecologists.” Diversity and Distributions 17: 43–57. 10.1111/j.1472-4642.2010.00725.x.

[ece374396-bib-0019] Erritzøe, J. , C. F. Mann , F. Brammer , and R. A. Fuller . 2012. Cuckoos of the World. Christopher Helm.

[ece374396-bib-0020] Evans, K. L. , K. J. Gaston , A. C. Frantz , et al. 2009. “Independent Colonization of Multiple Urban Centres by a Formerly Forest Specialist Bird Species.” Proceedings of the Royal Society B: Biological Sciences 276: 2403–2410. 10.1098/rspb.2008.1712. PMC2691005 19364751

[ece374396-bib-0021] Excoffier, L. , and H. E. Lischer . 2010. “Arlequin Suite Ver 3.5: A New Series of Programs to Perform Population Genetics Analyses Under Linux and Windows.” Molecular Ecology Resources 10: 564–567. 10.1111/j.1755-0998.2010.02847.x. 21565059

[ece374396-bib-0022] Falush, D. , M. Stephens , and J. K. Pritchard . 2003. “Inference of Population Structure Using Multilocus Genotype Data: Linked Loci and Correlated Allele Frequencies.” Genetics 164: 1567–1587. 10.1093/genetics/164.4.1567. PMC1462648 12930761

[ece374396-bib-0023] Feeney, W. E. , J. A. Welbergen , and N. E. Langmore . 2014. “Advances in the Study of Coevolution Between Avian Brood Parasites and Their Hosts.” Annual Review of Ecology, Evolution, and Systematics 45: 227–246. 10.1146/annurev-ecolsys-120213-091603.

[ece374396-bib-0024] Fick, S. E. , and R. J. Hijmans . 2017. “WorldClim 2: New 1‐Km Spatial Resolution Climate Surfaces for Global Land Areas.” International Journal of Climatology 37: 4302–4315. 10.1002/joc.5086.

[ece374396-bib-0025] Fossøy, F. , A. Antonov , A. Moksnes , et al. 2011. “Genetic Differentiation Among Sympatric Cuckoo Host Races: Males Matter.” Proceedings of the Royal Society B: Biological Sciences 278: 1639–1645. 10.1098/rspb.2010.2090. PMC3081775 21068043

[ece374396-bib-0026] Fossøy, F. , M. D. Sorenson , W. Liang , et al. 2016. “Ancient Origin and Maternal Inheritance of Blue Cuckoo Eggs.” Nature Communications 7: 10272. 10.1038/ncomms10272. PMC4729921 26754355

[ece374396-bib-0027] Friedmann, H. 1928. “The Origin of Host Specificity in the Parasitic Habit in the Cuculidae.” Auk 45: 33–38. 10.2307/4075354.

[ece374396-bib-0028] Fuisz, T. I. , and S. R. De Kort . 2007. “Habitat‐Dependent Call Divergence in the Common Cuckoo: Is It a Potential Signal for Assortative Mating?” Proceedings of the Royal Society B: Biological Sciences 274: 2093–2097. 10.1098/rspb.2007.0487. PMC2706194 17580296

[ece374396-bib-0029] Genovart, M. , J.‐C. Thibault , J. M. Igual , C. Bauzà‐Ribot , and V. Bretagnolle . 2013. “Population Structure and Dispersal Patterns Within and Between Atlantic and Mediterranean Populations of a Large‐Range Pelagic Seabird.” PLoS One 8: e70711. 10.1371/journal.pone.0070711. PMC3741395 23950986

[ece374396-bib-0030] Gibbs, H. L. , M. D. Sorenson , K. Marchetti , M. Brooke , N. Davies , and H. Nakamura . 2000. “Genetic Evidence for Female Host‐Specific Races of the Common Cuckoo.” Nature 407: 183–186. 10.1038/35025058. 11001055

[ece374396-bib-0031] Go, J.‐S. , H.‐N. Kim , S.‐J. Jin , M.‐C. Cha , H. Lee , and J.‐W. Lee . 2024. “Do Brood Parasitic Common Cuckoos Develop Brood Patches During the Breeding Season?” Avian Research 15: 100197. 10.1016/j.avrs.2024.100197.

[ece374396-bib-0032] Go, J.‐S. , J.‐W. Lee , and J.‐C. Yoo . 2021. “Variations of Hawk Mimicry Traits in the Four Sympatric *Cuculus* Cuckoos.” Frontiers in Ecology and Evolution 9: 702263. 10.3389/fevo.2021.702263.

[ece374396-bib-0033] Gower, J. C. 1966. “Some Distance Properties of Latent Root and Vector Methods Used in Multivariate Analysis.” Biometrika 53: 325–338. 10.1093/biomet/53.3-4.325.

[ece374396-bib-0034] Graham, B. A. , D. D. Heath , and D. J. Mennill . 2017. “Dispersal Influences Genetic and Acoustic Spatial Structure for Both Males and Females in a Tropical Songbird.” Ecology and Evolution 7: 10089–10102. 10.1002/ece3.3456. PMC5723598 29238539

[ece374396-bib-0035] Gregory, K. A. , C. Francesiaz , F. Jiguet , and A. Besnard . 2023. “A Synthesis of Recent Tools and Perspectives in Migratory Connectivity Studies.” Movement Ecology 11: 69. 10.1186/s40462-023-00388-z. PMC10605477 37891684

[ece374396-bib-0036] Hardy, O. J. , and X. Vekemans . 2002. “SPAGeDi: A Versatile Computer Program to Analyse Spatial Genetic Structure at the Individual or Population Levels.” Molecular Ecology Notes 2: 618–620. 10.1046/j.1471-8286.2002.00305.x.

[ece374396-bib-0037] Hewson, C. M. , K. Thorup , J. W. Pearce‐Higgins , and P. W. Atkinson . 2016. “Population Decline Is Linked to Migration Route in the Common Cuckoo.” Nature Communications 7: 12296. 10.1038/ncomms12296. PMC4960304 27433888

[ece374396-bib-0038] Jin, S.‐J. , H.‐N. Kim , J.‐S. Go , et al. 2025. “Vocal Variation in Female Common Cuckoos ( *Cuculus canorus* ): Geographic, Ecological and Morphological Perspectives.” Journal of Ornithology 167: 541–551. 10.1007/s10336-025-02347-4.

[ece374396-bib-0039] Jin, S.‐J. , J. W. Lee , and J. C. Yoo . 2025. “The Structural Function of the Bubbling Call of the Female Common Cuckoo ( *Cuculus canorus* ).” Ibis 167: 1018–1027. 10.1111/ibi.13412.

[ece374396-bib-0040] Jones, O. R. , and J. Wang . 2010. “COLONY: A Program for Parentage and Sibship Inference From Multilocus Genotype Data.” Molecular Ecology Resources 10: 551–555. 10.1111/j.1755-0998.2009.02787.x. 21565056

[ece374396-bib-0041] Koleček, J. , R. Piálková , L. Piálek , et al. 2021. “Spatiotemporal Patterns of Egg Laying in the Common Cuckoo.” Animal Behaviour 177: 107–116. 10.1016/j.anbehav.2021.04.021.

[ece374396-bib-0042] Koleček, J. , P. Procházka , V. Brlík , and M. Honza . 2020. “Cross‐Continental Test of Natal Philopatry and Habitat‐Imprinting Hypotheses to Explain Host Specificity in an Obligate Brood Parasite.” Science of Nature 107: 12. 10.1007/s00114-020-1667-0. 32108908

[ece374396-bib-0043] Lee, H. , H.‐N. Kim , J.‐S. Go , et al. 2023. “A Novel Method to Collect Sperm From Brood Parasitic Cuckoos: Urodeum Stimulation (UroS) Method.” Avian Research 14: 100085. 10.1016/j.avrs.2023.100085.

[ece374396-bib-0044] Lee, J.‐W. 2014. “Searching for Hosts of Avian Brood Parasites Breeding in Korea.” Korean Journal of Ornithology 21: 25–37.

[ece374396-bib-0045] Lee, J.‐W. , S.‐G. Kang , J.‐Y. Lee , et al. 2023. “Long‐Distance Migration of Korean Common Cuckoos With Different Host Specificities.” Global Ecology and Conservation 43: e02426. 10.1016/j.gecco.2023.e02426.

[ece374396-bib-0046] Lee, J.‐W. , D.‐W. Kim , and J.‐C. Yoo . 2005. “Egg Rejection by Both Male and Female Vinous‐Throated Parrotbills *Paradoxornis webbianus* .” Integrative Biosciences 9: 211–213. 10.1080/17386357.2005.9647273.

[ece374396-bib-0047] Lee, J.‐W. , H.‐K. Moon , H.‐J. Noh , M.‐S. Kim , and J.‐C. Yoo . 2021. “Host‐Dependent Dispersal Demonstrates Both‐Sex Host Specificity in Cuckoos.” Behavioral Ecology 32: 248–256. 10.1093/beheco/araa122.

[ece374396-bib-0048] Lee, J.‐W. , T. Simeoni , T. Burke , and B. Hatchwell . 2010. “The Consequences of Winter Flock Demography for Genetic Structure and Inbreeding Risk in Vinous‐Throated Parrotbills, *Paradoxornis webbianus* .” Heredity 104: 472–481. 10.1038/hdy.2009.135. 19812618

[ece374396-bib-0049] Lee, J.‐W. , and J.‐C. Yoo . 2004. “Effect of Host Egg Color Dimorphism on Interactions Between the Vinous‐Throated Parrotbill ( *Paradoxornis webbianus* ) and Common Cuckoo ( *Cuculus canorus* ).” Korean Journal of Biological Sciences 8: 77–80. 10.1080/12265071.2004.9647737.

[ece374396-bib-0050] Lee, S.‐i. , W. Kim , J. Choe , and M. Husby . 2016. “Genetic Assessment of the Subspecies Status of Eurasian Magpies ( *Pica pica* ) in Norway.” Ornis Fennica 93: 146–158. 10.51812/of.133897.

[ece374396-bib-0051] Leedale, A. E. , S. P. Sharp , M. Simeoni , E. J. Robinson , and B. J. Hatchwell . 2018. “Fine‐Scale Genetic Structure and Helping Decisions in a Cooperatively Breeding Bird.” Molecular Ecology 27: 1714–1726. 10.1111/mec.14553. 29543401

[ece374396-bib-0052] Lobo, J. M. , A. Jiménez‐Valverde , and R. Real . 2008. “AUC: A Misleading Measure of the Performance of Predictive Distribution Models.” Global Ecology and Biogeography 17: 145–151. 10.1111/j.1466-8238.2007.00358.x.

[ece374396-bib-0053] MacDougall‐Shackleton, E. A. , and S. A. MacDougall‐Shackleton . 2001. “Cultural and Genetic Evolution in Mountain White‐Crowned Sparrows: Song Dialects Are Associated With Population Structure.” Evolution 55: 2568–2575. 10.1111/j.0014-3820.2001.tb00769.x. 11831670

[ece374396-bib-0054] Maher, K. H. , L. J. Eberhart‐Phillips , A. Kosztolányi , et al. 2017. “High Fidelity: Extra‐Pair Fertilisations in Eight Charadrius Plover Species Are Not Associated With Parental Relatedness or Social Mating System.” Journal of Avian Biology 48: 910–920. 10.1111/jav.01263.

[ece374396-bib-0055] Marchetti, K. , H. Nakamura , and H. L. Gibbs . 1998. “Host‐Race Formation in the Common Cuckoo.” Science 282: 471–472. 10.1126/science.282.5388.471. 9774273

[ece374396-bib-0056] McPherson, J. M. , and W. Jetz . 2007. “Effects of Species' Ecology on the Accuracy of Distribution Models.” Ecography 30: 135–151. 10.1111/j.0906-7590.2007.04823.x.

[ece374396-bib-0057] Medina, I. , and N. E. Langmore . 2016. “The Evolution of Host Specialisation in Avian Brood Parasites.” Ecology Letters 19: 1110–1118. 10.1111/ele.12649. 27417381

[ece374396-bib-0058] Merondun, J. , F. Fossøy , S. Meshcheryagina , et al. 2025. “Genomic Architecture of Egg Mimicry and Its Consequences for Speciation in Parasitic Cuckoos.” Science 390: 527–532. 10.1126/science.adt9355. 41166466

[ece374396-bib-0059] Merondun, J. , C. I. Marques , P. Andrade , et al. 2024. “Evolution and Genetic Architecture of Sex‐Limited Polymorphism in Cuckoos.” Science Advances 10: eadl5255. 10.1126/sciadv.adl5255. PMC11042743 38657058

[ece374396-bib-0060] Merow, C. , and J. A. Silander . 2014. “A Comparison of M Axlike and M Axent for Modelling Species Distributions.” Methods in Ecology and Evolution 5: 215–225.

[ece374396-bib-0061] Merow, C. , M. J. Smith , and J. A. Silander Jr. 2013. “A Practical Guide to MaxEnt for Modeling Species' Distributions: What It Does, and Why Inputs and Settings Matter.” Ecography 36: 1058–1069. 10.1111/j.1600-0587.2013.07872.x.

[ece374396-bib-0062] Miller, M. P. , T. D. Mullins , J. W. Parrish Jr. , J. R. Walters , and S. M. Haig . 2012. “Variation in Migratory Behavior Influences Regional Genetic Diversity and Structure Among American Kestrel Populations ( *Falco sparverius* ) in North America.” Journal of Heredity 103: 503–514. 10.1093/jhered/ess024. 22563129

[ece374396-bib-0063] Moksnes, A. , and E. Røskaft . 1995. “Egg‐Morphs and Host Preference in the Common Cuckoo ( *Cuculus canorus* ): An Analysis of Cuckoo and Host Eggs From European Museum Collections.” Journal of Zoology 236: 625–648. 10.1111/j.1469-7998.1995.tb02736.x.

[ece374396-bib-0064] Moskát, C. , M. Bán , A. Fülöp , J. Bereczki , and M. E. Hauber . 2019. “Bimodal Habitat Use in Brood Parasitic Common Cuckoos ( *Cuculus canorus* ) Revealed by GPS Telemetry.” Auk: Ornithological Advances 136: 1–12. 10.1093/auk/uky019.

[ece374396-bib-0065] National Institute of Ecology . 2024. The 5th National Ecosystem Survey (2019–2023). National Institute of Ecology.

[ece374396-bib-0066] Payne, R. B. , and M. D. Sorensen . 2005. The Cuckoos. Oxford University Press.

[ece374396-bib-0067] Peterman, W. E. , and N. S. Pope . 2021. “The Use and Misuse of Regression Models in Landscape Genetic Analyses.” Molecular Ecology 30: 37–47. 10.1111/mec.15716. 33128830

[ece374396-bib-0068] Phillips, S. J. , R. P. Anderson , and R. E. Schapire . 2006. “Maximum Entropy Modeling of Species Geographic Distributions.” Ecological Modelling 190: 231–259. 10.1016/j.ecolmodel.2005.03.026.

[ece374396-bib-0069] Phillips, S. J. , and M. Dudík . 2008. “Modeling of Species Distributions With Maxent: New Extensions and a Comprehensive Evaluation.” Ecography 31: 161–175. 10.1111/j.0906-7590.2008.5203.x.

[ece374396-bib-0070] Pritchard, J. K. , M. Stephens , and P. Donnelly . 2000. “Inference of Population Structure Using Multilocus Genotype Data.” Genetics 155: 945–959. 10.1093/genetics/155.2.945. PMC1461096 10835412

[ece374396-bib-0071] QGIS Development Team . 2026. QGIS Geographic Information System. QGIS Association.

[ece374396-bib-0072] Queller, D. C. , and K. F. Goodnight . 1989. “Estimating Relatedness Using Genetic Markers.” Evolution 43: 258–275. 10.1111/j.1558-5646.1989.tb04226.x. 28568555

[ece374396-bib-0073] R Core Team . 2024. R: A Language and Environment for Statistical Computing. R Foundation for Statistical Computing.

[ece374396-bib-0074] Rothstein, S. I. 1990. “A Model System for Coevolution: Avian Brood Parasitism.” Annual Review of Ecology and Systematics 21: 481–508. 10.1146/annurev.es.21.110190.002405.

[ece374396-bib-0075] Rousset, F. 2008. “genepop'007: A Complete Re‐Implementation of the Genepop Software for Windows and Linux.” Molecular Ecology Resources 8: 103–106. 10.1111/j.1471-8286.2007.01931.x. 21585727

[ece374396-bib-0076] Row, J. R. , S. T. Knick , S. J. Oyler‐McCance , S. C. Lougheed , and B. C. Fedy . 2017. “Developing Approaches for Linear Mixed Modeling in Landscape Genetics Through Landscape‐Directed Dispersal Simulations.” Ecology and Evolution 7: 3751–3761. 10.1002/ece3.2825. PMC5468135 28616172

[ece374396-bib-0077] Smouse, P. E. , and R. Peakall . 1999. “Spatial Autocorrelation Analysis of Individual Multiallele and Multilocus Genetic Structure.” Heredity 82: 561–573. 10.1038/sj.hdy.6885180. 10383677

[ece374396-bib-0078] Sokolov, L. , A. Y. Sinelschikova , and M. Y. Markovets . 2024. “The First Satellite Tracking Data on the Migration of the Common Cuckoo *Cuculus canorus* (Cuculiformes, Cuculidae) From Southern Siberia (Khakassia, Russia).” Proceedings of the Zoological Institute RAS 328: 374–378. 10.31610/trudyzin/2024.328.3.374.

[ece374396-bib-0777] Sorenson, M. , K. Sefc , and R. Payne . 2003. “Speciation by Host Switch in Brood Parasitic Indigobirds.” Nature 424: 928–931. 10.1038/nature01863. 12931185

[ece374396-bib-0079] Šulc, M. , A. E. Hughes , J. Troscianko , et al. 2022. “Automatic Identification of Bird Females Using Egg Phenotype.” Zoological Journal of the Linnean Society 195: 33–44. 10.1093/zoolinnean/zlab051.

[ece374396-bib-0080] Swets, J. A. 1988. “Measuring the Accuracy of Diagnostic Systems.” Science 240: 1285–1293. 10.1126/science.3287615. 3287615

[ece374396-bib-0081] Teuschl, Y. , B. Taborsky , and M. Taborsky . 1998. “How Do Cuckoos Find Their Hosts? The Role of Habitat Imprinting.” Animal Behaviour 56: 1425–1433. 10.1006/anbe.1998.0931. 9933539

[ece374396-bib-0082] Uy, J. A. C. , D. E. Irwin , and M. S. Webster . 2018. “Behavioral Isolation and Incipient Speciation in Birds.” Annual Review of Ecology, Evolution, and Systematics 49: 1–24. 10.1146/annurev-ecolsys-110617-062646.

[ece374396-bib-0083] Van Oosterhout, C. , W. F. Hutchinson , D. P. Wills , and P. Shipley . 2004. “MICRO‐CHECKER: Software for Identifying and Correcting Genotyping Errors in Microsatellite Data.” Molecular Ecology Notes 4: 535–538. 10.1111/j.1471-8286.2004.00684.x.

[ece374396-bib-0084] Vega, M. L. , M. Willemoes , R. L. Thomson , et al. 2016. “First‐Time Migration in Juvenile Common Cuckoos Documented by Satellite Tracking.” PLoS One 11: e0168940. 10.1371/journal.pone.0168940. PMC5179092 28005960

[ece374396-bib-0085] Vogl, W. , M. Taborsky , B. Taborsky , Y. Teuschl , and M. Honza . 2002. “Cuckoo Females Preferentially Use Specific Habitats When Searching for Host Nests.” Animal Behaviour 64: 843–850. 10.1006/anbe.2003.1967.

[ece374396-bib-0086] Willemoes, M. , R. Strandberg , R. H. Klaassen , et al. 2014. “Narrow‐Front Loop Migration in a Population of the Common Cuckoo *Cuculus canorus* , as Revealed by Satellite Telemetry.” PLoS One 9: e83515. 10.1371/journal.pone.0083515. PMC3885432 24421890

[ece374396-bib-0087] Williams, H. M. , M. Willemoes , R. H. Klaassen , R. Strandberg , and K. Thorup . 2016. “Common Cuckoo Home Ranges Are Larger in the Breeding Season Than in the Non‐Breeding Season and in Regions of Sparse Forest Cover.” Journal of Ornithology 157: 461–469. 10.1007/s10336-015-1308-0.

[ece374396-bib-0088] Wright, S. 1949. “The Genetical Structure of Populations.” Annals of Eugenics 15: 323–354. 10.1111/j.1469-1809.1949.tb02451.x. 24540312

[ece374396-bib-0089] Wright, T. F. , A. M. Rodriguez , and R. C. Fleischer . 2005. “Vocal Dialects, Sex‐Biased Dispersal, and Microsatellite Population Structure in the Parrot *Amazona auropalliata* .” Molecular Ecology 14: 1197–1205. 10.1111/j.1365-294X.2005.02466.x. 15773946

[ece374396-bib-0090] Yang, C. , W. Liang , and A. P. Møller . 2018. “Do Cuckoos Imprint on Hosts, Micro‐Habitats, or Nest Sites? Parasitism Preferences in the Common Cuckoo ( *Cuculus canorus* ).” Behavioral Ecology and Sociobiology 72: 126. 10.1007/s00265-018-2542-2.

[ece374396-bib-0091] Zanaga, D. , R. Van De Kerchove , D. Daems , et al. 2022. “ESA WorldCover 10 m 2021 v200.” 10.5281/zenodo.7254221.

