# Supplementary material for: Ecological Context Constrains Host‐Associated Genetic Structure in the Common Cuckoo
Source: Ecol Evol. 2026 Sep 17;16(9):e74396. doi: 10.1002/ece3.74396 (PMC13585489; doi:10.1002/ece3.74396)
Supplement: Supplementary file 1 [file ECE3-16-e74396-s003.docx]

Table S1. Summary statistics and quality-control tests for 11 microsatellite loci screened in the common cuckoo. Results of Hardy–Weinberg equilibrium (HWE) and linkage disequilibrium (LD) tests are shown for the 11 microsatellite loci initially screened in the common cuckoo, together with the number of alleles per locus (N_A_), expected heterozygosity (H_E_), and within-population inbreeding coefficient (F_IS_). Loci showing significant deviations from HWE are indicated by asterisks.

| Locus | HWE | LD | N_A_ | H_E_ | F_IS_ |
| --- | --- | --- | --- | --- | --- |
| Ccμ01 | NS | NS | 3 | 0.0791 | 0.0276 |
| Ccu09 | NS | NS | 4 | 0.0432 | -0.0167 |
| Ccμ13 | NS | NS | 7 | 0.6187 | -0.0169 |
| Ccμ60 | * | NS | 16 | 0.8705 | 0.0911 |
| Ccμ88 | NS | NS | 8 | 0.8298 | -0.0164 |
| Ccμ100 | NS | NS | 9 | 0.8059 | 0.0497 |
| Ccμ108 | NS | NS | 6 | 0.6630 | 0.0719 |
| Ccμ119 | NS | NS | 22 | 0.9172 | 0.0209 |
| Ccμ137 | NS | NS | 19 | 0.8518 | 0.0472 |
| Clu03 | * | NS | 10 | 0.5095 | 0.0942 |
| Clu05 | * | NS | 12 | 0.7934 | 0.1240 |

NS, not significant; * *p* < 0.01.

Table S2. Definitions of the seven bioclimatic variables (WorldClim) used in this study. Variables were selected to minimize collinearity while capturing temperature and precipitation gradients relevant to the study region.

| **Variable** | **Description** | **Calculation** | **Unit** |
| --- | --- | --- | --- |
| BIO2 | Mean diurnal range | Mean of monthly (max temp − min temp) | °C |
| BIO3 | Isothermality | (BIO2 / BIO7) × 100 | % |
| BIO4 | Temperature seasonality | Standard deviation of monthly mean temperature × 100 | — |
| BIO10 | Mean temperature of warmest quarter | Mean temperature of the warmest quarter (three warmest consecutive months) | °C |
| BIO12 | Annual precipitation | Sum of monthly precipitation over the year | mm |
| BIO14 | Precipitation of driest month | Precipitation of the driest month (minimum monthly precipitation) | mm |
| BIO16 | Precipitation of wettest quarter | Total precipitation of the wettest quarter (three wettest consecutive months) | mm |

*Note. BIO3 (isothermality) is derived from BIO7, the temperature annual range, defined as BIO5 and BIO6, where BIO5 is the maximum temperature of the warmest month and BIO6 is the minimum temperature of the coldest month (all in °C). BIO7, BIO5, and BIO6 were used only to compute BIO3 and were not themselves included as predictors.*

**Figure S1.** STRUCTURE analysis of 364 common cuckoos (*Cuculus canorus*) genotyped at 10 microsatellite loci. (a) Mean log-likelihood of the data [Ln P(D)] as a function of the assumed number of clusters (K = 1–16). (b, c) Individual admixture proportions at K = 2 and K = 3, respectively. Each vertical bar represents one individual, and individuals are grouped by sampling population.

Figure S2. Observed occurrence and predicted distribution of the meadow bunting (*Emberiza cioides*) in South Korea. (a) Compiled occurrence records (n = 1,396) used for species distribution modelling. (b) Predicted habitat suitability from a Maxent model (mean of 10 replicate runs; values range from 0 = unsuitable to 1 = highly suitable). yellow colors indicate higher predicted suitability, whereas blue colors indicate lower predicted suitability.
